# Supplementary material for: Effects of day-to-day variation of Opisthorchis viverrini antigen in urine on the accuracy of diagnosing opisthorchiasis in Northeast Thailand
Source: PLoS One. 2022 Jul 19;17(7):e0271553. doi: 10.1371/journal.pone.0271553 (PMC9295949; doi:10.1371/journal.pone.0271553)
Supplement: S1 Table — (DOCX) [file pone.0271553.s004.docx]

**S1 Table.** **Baseline prevalence of parasitic infection among project participants (*n*=1,471) as determined by FECT and stratified by locality (Muang District, Khon Kaen Province (KKN, *n*=448) and Nong Kung Sri District, Kalasin Province (KSN, *n*=1,023).**

| **Variable** | **Locality** | | **Total** |
| --- | --- | --- | --- |
|  | **KKN** | **KSN** |  |
| **No. of participants** | 448 (%) | 1,023 (%) | 1,471 (%) |
| **Parasite** |  |  |  |
| *Opisthorchis viverrini* | 91 (20.3) | 108 (10.6) | 199 (13.5) |
| *Strongyloides stercoralis* | 48 (10.7) | 124 (12.1) | 172 (11.7) |
| Minute intestinal flukes | 11 (2.3) | 70 (6.8) | 81 (5.5) |
| Hookworm | 5 (1.1) | 6 (0.6) | 11 (0.7) |
| *Taenia* sp. | 4 (0.9) | 18 (1.8) | 22 (1.5) |
| *Echinostoma* sp. | 5 (1.1) | 61 (6.0) | 66 (4.5) |
| *Trichuris trichiura* | 0 (0) | 1 (0.01) | 1 (0.07) |
| *Hymenolepis diminuta* | 0 (0) | 1 (0.01) | 1 (0.07) |
| *Enterobius vermicularis* | 0 (0) | 5 (0.5) | 5 (0.34) |
| *Giardia lamblia* | 0 (0) | 8 (0.8) | 8 (0.5) |
| *Blastocystis hominis* | 11 (2.5) | 12 (1.2) | 23 (1.6) |
