# Supplementary material for: Effects of day-to-day variation of Opisthorchis viverrini antigen in urine on the accuracy of diagnosing opisthorchiasis in Northeast Thailand
Source: PLoS One. 2022 Jul 19;17(7):e0271553. doi: 10.1371/journal.pone.0271553 (PMC9295949; doi:10.1371/journal.pone.0271553)
Supplement: S2 Table — (DOCX) [file pone.0271553.s005.docx]

**S2 Table. The age- and sex-prevalence of *O. viverrini* infection determined by FECT and urine assay in Muang District, Khon Kaen Province (KKN), Nong Kung Sri District, Kalasin Province (KSN) (*n*=1,471).**

| **Site** | **Variable** | **N** | **Positive by FECT  n (%)^(a)^** | **Positive by**  **urine assay  n (%)^(a)^** | **P-value**  **(X^2^-test)** |
| --- | --- | --- | --- | --- | --- |
| **KKN** | **Age group** |  |  |  |  |
|  | <40 | 52 | 4 (7.7) | 4 (7.7) | > 0.05 |
|  | <50 | 92 | 11 (12.0) | 15 (16.3) | > 0.05 |
|  | <60 | 149 | 39 (26.2) | 37 (24.8) | > 0.05 |
|  | >60 | 155 | 37 (23.8) | 36 (23.2) | > 0.05 |
|  | **Sex** |  |  |  |  |
|  | Male | 186 | 36 (19.4) | 34 (18.3) | > 0.05 |
|  | Female | 262 | 55 (21.0) | 58 (22.1) | > 0.05 |
|  | **Total** | 448 | 91(20.3) | 92 (20.5) | > 0.05 |
| **KSN** | **Age group** |  |  |  |  |
|  | <40 | 127 | 8 (6.3) | 26 (20.5) | < 0.001 |
|  | <50 | 337 | 32 (9.5) | 86 (25.5) | < 0.001 |
|  | <60 | 337 | 33 (9.8) | 92 (27.3) | < 0.001 |
|  | >60 | 222 | 35 (15.8) | 78 (35.1) | < 0.001 |
|  | **Sex** |  |  |  |  |
|  | Male | 497 | 52 (10.5) | 152 (30.6) | < 0.001 |
|  | Female | 526 | 56 (10.6) | 130 (24.7) | < 0.001 |
|  | **Total** | 1,023 | 108 (10.5) | 282 (27.5) | < 0.001 |

1. Significant association with age p < 0.05, X^2^-test = chi-square test
