# Supplementary material for: Effects of day-to-day variation of Opisthorchis viverrini antigen in urine on the accuracy of diagnosing opisthorchiasis in Northeast Thailand
Source: PLoS One. 2022 Jul 19;17(7):e0271553. doi: 10.1371/journal.pone.0271553 (PMC9295949; doi:10.1371/journal.pone.0271553)
Supplement: S3 Table — Analyses using linear regression models were performed based on each locality (KKN and KSN) separately and combined. (DOCX) [file pone.0271553.s006.docx]

**S3 Table. Relationships between antigen concentrations of *O. viverrini* and intensity of infection (EPG) determined by FECT. Analyses using linear regression models were performed based on each locality (KKN and KSN) separately and combined.**

| **Locality** | **Equation** | **R^2^** | **P-value** |
| --- | --- | --- | --- |
| KKN | y = 0.253x + 1.211 | 0.551 | < 0.001 |
| KSN | y = 0.159x + 1.229 | 0.153 | < 0.001 |
| Both sites | y = 0.156x + 1.219 | 0.155 | < 0.001 |

KKN: Muang district, Khon Kaen Province, KSN: Nong Kung Sri district, Kalasin Province. R^2^ = the coefficient of determination.
