# Supplementary material for: Effects of day-to-day variation of Opisthorchis viverrini antigen in urine on the accuracy of diagnosing opisthorchiasis in Northeast Thailand
Source: PLoS One. 2022 Jul 19;17(7):e0271553. doi: 10.1371/journal.pone.0271553 (PMC9295949; doi:10.1371/journal.pone.0271553)
Supplement: S4 Table — KKN: Muang District, Khon Kaen Province, KSN: Nong Kung Sri District, Kalasin Province. (DOCX) [file pone.0271553.s007.docx]

**S4 Table. Positive rates of *O. viverrini* infection determined by urine antigen detection assay in groups of participants stratified by intensity of infection (EPG). KKN: Muang District, Khon Kaen Province, KSN: Nong Kung Sri District, Kalasin Province.**

| **Site** | **Intensity group**  **(*O. viverrini*** **EPG)** | **N** | **Number positive by urine assay (%)** |
| --- | --- | --- | --- |
| KKN | 0 | 183 | 7 (3.8) |
|  | Low EPG (1-50) | 56 | 50 (89.9) |
|  | Moderate EPG (51-100) | 9 | 9 (100) |
|  | High EPG (>100) | 6 | 6 (100) |
|  | **Total** | **254** | **72 (28.3)** |
| KSN | 0 | 456 | 91 (20.0) |
|  | Low EPG (1-50) | 69 | 58 (84.1) |
|  | Moderate EPG (51-100) | 12 | 11 (91.6) |
|  | High EPG (>100) | 10 | 10 (100) |
|  | **Total** | **547** | **170 (31.1)** |
| Both sites | 0 | 639 | 98 (15.3) |
|  | Low EPG (1-50) | 125 | 108 (86.4) |
|  | Moderate EPG (51-100) | 21 | 20 (95.2) |
|  | High EPG (>100) | 16 | 16 (100) |
|  | **Total** | **801** | **242 (30.2)** |
